# Structural and Functional Restraints on the Occurrence of Single Amino Acid Variations in Human Proteins

**DOI:** 10.1371/journal.pone.0009186

**Published:** 2010-02-12

**Authors:** Sungsam Gong, Tom L. Blundell

**Affiliations:** Biocomputing Group, Department of Biochemistry, University of Cambridge, Cambridge, United Kingdom; Leeds Institute of Molecular Medicine, United Kingdom

## Abstract

Human genetic variation is the incarnation of diverse evolutionary history, which reflects both selectively advantageous and selectively neutral change. In this study, we catalogue structural and functional features of proteins that restrain genetic variation leading to single amino acid substitutions. Our variation dataset is divided into three categories: i) Mendelian disease-related variants, ii) neutral polymorphisms and iii) cancer somatic mutations. We characterize structural environments of the amino acid variants by the following properties: i) side-chain solvent accessibility, ii) main-chain secondary structure, and iii) hydrogen bonds from a side chain to a main chain or other side chains. To address functional restraints, amino acid substitutions in proteins are examined to see whether they are located at functionally important sites involved in protein-protein interactions, protein-ligand interactions or catalytic activity of enzymes. We also measure the likelihood of amino acid substitutions and the degree of residue conservation where variants occur. We show that various types of variants are under different degrees of structural and functional restraints, which affect their occurrence in human proteome.

## Introduction

The evolution of orthologous proteins occurs through the establishment of amino acid substitutions in the population at rates that depend on restraints arising from the need to maintain proper three-dimensional structure and to retain functional interactions of each amino acid within or between molecules [1]–[4]. For example, amino acids in the cores of proteins are relatively conserved compared with those in the solvent accessible regions [5], [6] and catalytic amino acids responsible for enzymatic reaction are also well conserved throughout evolution. Hence, mutations tend to be accepted in amino acid residues where evolutionary pressure is relatively relaxed and where they can remain in the population without selective disadvantage (or advantage). Recently, high-throughput DNA sequencing technology has begun to have a major impact on this field and is shedding light on genomic sequence variations between human individuals [7]–[10]. Single nucleotide polymorphisms (SNPs) in protein coding regions are of special interest as they may be non-synonymous (nsSNPs), resulting in changes in the types of amino acid in the protein products. Indeed, recent analysis of human nsSNPs shows that the majority are commonly found and appear to be functionally neutral [11]. Thus, it is of interest to examine whether the occurrence of coding variations in the human population is equally affected by the factors that restrain the substitutions of amino acids observed in divergent evolution of proteins.

Before the determination of the human genome sequence, analysis of genetic mutations focused on establishing the relationship between genotypes and their phenotypes, especially susceptibility to certain disease types [12], [13]. Detailed molecular analyses of protein structure and function have shown that single amino acid substitutions or mutations are often responsible for certain disease types [14], [15]. It has been claimed that ∼60% of such Mendelian disease mutations arise from amino acid substitutions in their respective genes (see [16] for review). For most monogenic diseases, a single DNA variant resulting in an amino acid substitution is responsible for a certain disease type by affecting protein stability and thus function [17]. Hence, much effort has been expended to characterize the pattern of nsSNPs in the context of sequences and structures of proteins in attempts establish whether they are likely to be neutral or deleterious for the functions of the organism [18]–[20].

One of the consensus agreements from molecular analyses of coding variants is that, although most of them are selectively neutral, their occurrence is restrained by various factors such as solvent accessibility, type of secondary structure, and presence of side-chain hydrogen bonding. Compared with benign and neutral variants, disease-related variants are more likely to be located in solvent inaccessible regions and tend to change the physicochemical properties from those of the wild type amino acids [14], [18]. In addition, disease-related variants are more likely to be located at conserved residues, which are believed to be functionally important [21], [22]. However, previous analyses have been based on relatively small sub-sets of sequence variants, and have not fully taken advantage of the rapid growing information on protein structure and function. Hence, in the era of information deluge from high-speed genome sequencing, high-resolution protein structure determination, and enriched annotation on protein functions, it is desirable to have large-scale cataloguing of coding variants in the light of structure and function of proteins. This will help us understand not only the nature of deleterious mutations, but also the evolutionary nature of the occurrence of single amino acid variations.

In this report, we address structural and functional restraints that shape the occurrence of single amino acid variations. We divide our data into three categories: i) Mendelian disease-related variants, ii) neutral polymorphisms and iii) cancer somatic mutations. We further characterize structural environments of amino acid variants by mapping sequence positions onto their corresponding three-dimensional structures if available. We observe, as reported previously [14], [18], that nsSNPs occur less frequently at the solvent inaccessible region of proteins, whereas disease-related mutations occur much more frequently than the average. We also find that cancer somatic mutations and disease-related variants occur more frequently at amino acids making hydrogen bonds from side chains than neutral polymorphisms. We measure substitution scores and the degree of sequence conservation at the variant positions and compare their differences by datasets.

## Results and Discussion

### Compilation of Amino Acid Variant Dataset

We compiled our variant dataset from the following sources: 1) Swiss-Prot human variants [23], 2) Ensembl human variation database [24], and 3) COSMIC (Catalogue Of Somatic Mutation In Cancer) database [25] (see Materials and Methods for details). The Swiss-Prot variants are further classified by Mendelian disease-related variants (SVD) and polymorphic variants (SVP) according to the original annotations from the source. For Ensembl human variations (SAP), we used only verified SNPs in order to ensure an accurate and reliable polymorphic dataset. The COSMIC dataset (CSM) differs from the others in that it contains somatic mutations observed in various cancer types. The sequence positions of variants from the source data were transferred to UniProt protein sequence level [26] and further mapped onto their corresponding locations in terms of three-dimensional structures if available in PDB [27]. Table 1 shows the number of variants from the source data, variants mapped onto UniProt protein level, and PDB level. SVD does not share variants with SVP, but does share 232 and 104 variants with CSM and SAP respectively, which are less than 1.4% of SVD (see Figure S1 for details). CSM shares less than 0.9% either with SAP (15/4476) or SVP (31/4476). However, SVP and SAP share ∼51% (16863/32748) and ∼57% (16863/29541) with each other, which is not surprising because both represent polymorphic variants. Considering the low percentage of overlaps amongst Mendelian disease (SVD), cancer somatic (CSM) and neutral polymorphic variants (SAP and SVP), we did not remove overlaps in our analysis, which we now describe.

**Table 1:** Four types of sequence variants and their numbers.

Sources	Types	Abbreviations	NO. of distinct variants		
			from the source	mapped to UniProt	mapped to PDB
UniProt	disease	SVD	16,776	16,776	4,942
	polymorphism	SVP	32,748	32,748	2,895
Ensembl	verified SNPs	SAP	29,541	28,702	2,024
COSMIC	cancer mutations	CSM	5,260	4,476	2,016

### Local Structural Environments of Sequence Variants

We wish to characterize the local structural environments of amino acid variants where three-dimensional structures of proteins are known. The local structural environments of amino acids were first defined as suggested by Overington and colleagues [28], [29]: 1) main-chain conformation and secondary structure, 2) solvent accessibility and 3) hydrogen bonding between side chains and main chains. In this framework, there could be 64 distinct environments for a residue from the combination of structural features: four from secondary structures (α-helix, β-strand, coil and residue with positive ϕ main-chain torsion angle), two from solvent accessibility (accessible and inaccessible), and eight (2^3^) from hydrogen bonds to main-chain carbonyl (CO) or amide (NH) or to another side chain. Four types of variants were mapped onto PDB structures and characterized by their local structural environments (see Datasets S1, S3 and S5). In Table 2, we quantified the proportions of variants that belong to each environmental category and compared them among four variant classes. To give background proportions of amino acids for each environmental feature, we counted amino acids from representative domains (see Materials and Methods) of SCOP families [30] and their proportions are given in Table 2. We investigated whether the ratio of variants for each environment category could result from the structural restraints that shape the occurrence of variants in proteins.

**Table 2:** Occurrence (%) of variants by structural environments.

Structural environment			Types of variants				Background
Categories		types	SVD^7^	SVP^8^	CSM^9^	SAP^10^	SCOP^11^
solvent accessibility		a^1^	42.25	18.45	26.45	19.48	31.21
hydrogen bonds from side chains	to main-chain amides	T^2^	10.69	5.79	8.44	5.69	8.55
	to main-chain carbonyls	T	19.50	13.01	13.27	13.36	13.63
	to other side chains	T	25.58	19.31	21.93	17.04	19.97
secondary structure		H^3^	27.98	32.98	22.14	31.58	36.61
		E^4^	23.25	20.23	20.26	20.13	21.09
		P^5^	9.71	6.40	10.26	6.60	6.45
		C^6^	39.06	40.39	47.34	41.69	35.85

1 : inaccessible.

2 : True (hydrogen bonded).

3 : α-helix.

4 : β-strand.

5 : positive ϕ main-chain torsion angle.

6 : coil.

7 : see Dataset S1.

8 : see Dataset S3.

9 : see Dataset S7.

10 : see Dataset S5.

11 : see ‘Representative SCOP domains’ of Materials and Methods.

#### By solvent accessibility

We observed that Mendelian disease-related variants (SVD) occur twice as often as polymorphic variants (SVP and SAP) at solvent inaccessible positions. For cancer mutations (CSM), the proportion of variants in solvent inaccessible regions is more than that of SVP but less than SVD. If a sequence variant occurs randomly in proteins, the probability of being located in a solvent inaccessible region would be close to 31.21%, which is the proportion of solvent inaccessible amino acids from the representative SCOP domains. As shown in Table 2, SVD occur 35% (42.25/31.21 -1) more than expected, whereas polymorphic variants (SVP and SAP) occur 40% (1 - 18.45/31.21) less often than expected. We presume that the differences in the frequency of occurrence by mutation types may arise from evolutionary pressure, which restricts the occurrence of variants in the core regions of proteins in order to minimize the effects on the stabilities of proteins. This observation also agrees with the finding that for most monogenic diseases a single DNA variant, resulting in an amino acid substitution, is responsible for the disease by affecting protein stability [17].

#### By hydrogen-bond capacity

For three categories of hydrogen-bond types, SVD occur more frequently at amino acids making hydrogen bonds (‘T’ in Table 2) than do the other variants. CSM also occur more frequently than polymorphic variants, but the difference is smaller than that of SVD. This observation, together with the ratios of occurrence in the interior/surface regions of proteins, clearly shows that amino acid variants are under strong restraints, resulting in the observation that they occur less frequently in regions maintaining the architectures of protein structures.

#### By element of secondary structure

As shown in Table 2, compared with the ratios of residues from representative SCOP domains and other polymorphic variants (SVP and SAP), SVD and CSM occur less in residues in α-helices (H), but more often at residues with positive ϕ main-chain torsion angles (P). Interestingly, almost half of CSM (47.34%) occur in coil regions, distinguishing them from other variant datasets (∼41.69%). Our results agree with those of Ferrer-Costa and colleagues [18] who showed disease-related SNPs occur less in α-helices but more frequently in β-strands than neutral nsSNPs, although differences in the percentages may arise from the methods used for defining secondary structure.

### Amino Acids Substitution Scores

**Figure 1:**
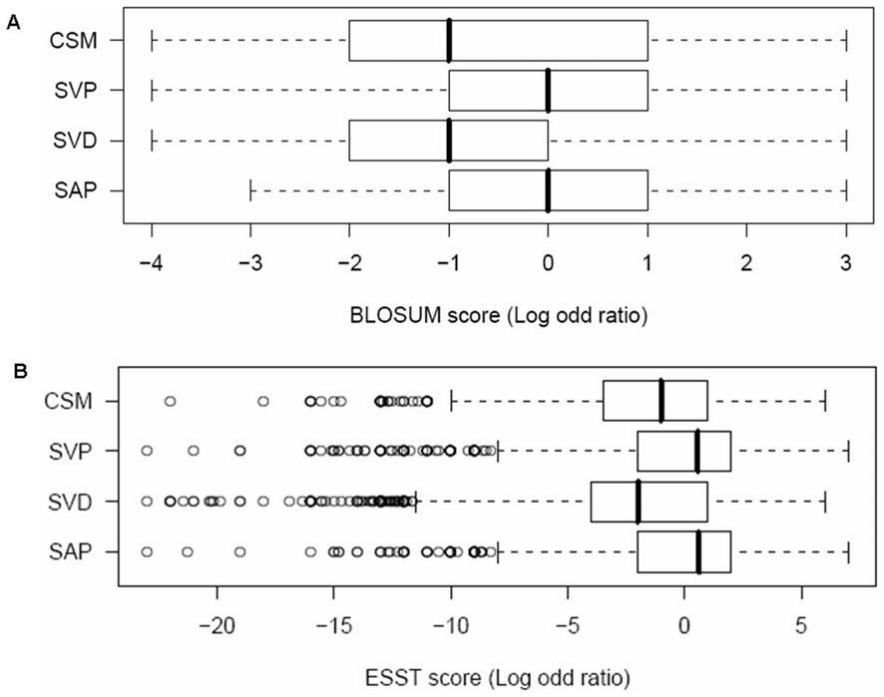
Box plots of substitution scores from four types of variants in the dataset. Each box plot is derived from the four variant datasets (see Table 1) and data are plotted against the BLOSUM62 substitution table and ESST in A and B, respectively. The median value is represented as a bold vertical line within a box, which represents the interquartile range (IQR) where lower quartile (cut-off at the lowest 25% of the data) and upper quartile (cut-off at the highest 25% of the data) are the left and right edges of the box. Two vertical lines extended from the left and right hand sides of a box represent the smallest (left whisker) and largest (right whisker) non-outlier observations, respectively. Any data observation that lies more than 1.5*IQR lower than the lower quartile or 1.5*IQR higher than the upper quartile is considered an outlier which is shown as a circle.

Amino acid substitution models such as PAM [31] and BLOSUM [32] describe the degree of substitutions as log-odd ratio values where the positive scores suggest commonly occurring and preferred substitutions, whereas the negative scores imply very rare substitutions which are disfavoured in nature. Those substitution tables were widely used to assess and predict the effects of nsSNPs [12], [18]. An ESST (Environment Specific Substitution Table, http://www-cryst.bioc.cam.ac.uk/esst) also describes the degree of substitution of amino acids, but differs from PAM or BLOSUM by taking into account structural environments which restrict the possible and allowable substitutions [28], [29]. Hence, ESSTs provide more accurate and discriminating measures of substitution probabilities in a particular environment in a three-dimensional protein structure. Figures 1A and 1B show box plots of substitution scores from four types of variants in the dataset using BLOSUM62 and ESST, respectively. From both models, the median substitution scores for SVD and CSM are lower than those of SVP and SAP. We further investigated substitution scores by the local structural environments of the variants where they occur in three-dimensional structures of proteins.

#### By solvent accessibility

**Figure 2:**
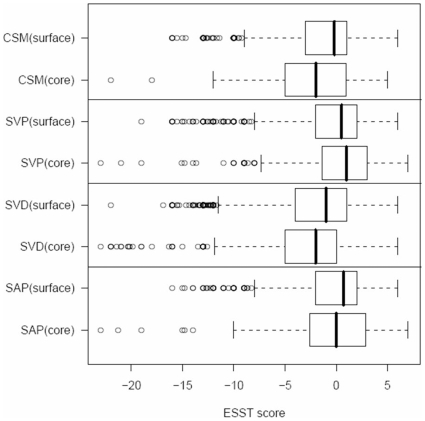
Box plots of substitution scores by solvent accessibility. Each of the four datasets is divided into solvent accessible (surface) and inaccessible (core) datasets. The representation scheme of a box plot is the same as shown in Figure 1.

Figure 2 shows box plots of substitution scores by solvent accessibility for the four types of variant dataset. Except for SVP, the median values of substitution scores in the core regions of proteins are always smaller than those from the surface regions. The difference in substitution scores between core and surface region is highly significant for both SVD and CSM (*P*<1.0^−12^) and significant for SVP (*P*<1.0^−4^), whereas it is not significant for SAP (*P*<0.78). This suggests that, although variants occur less frequently at solvent inaccessible regions, their effect would be detrimental if they occurred at the solvent inaccessible regions. In addition, the average proportions of variants having negative values of substitution score are 63% and 55% for SVD and CSM respectively, whereas the average proportions are less than 40% for SVP and SAP (see Table S1).

#### By hydrogen-bond capacity

**Figure 3:**
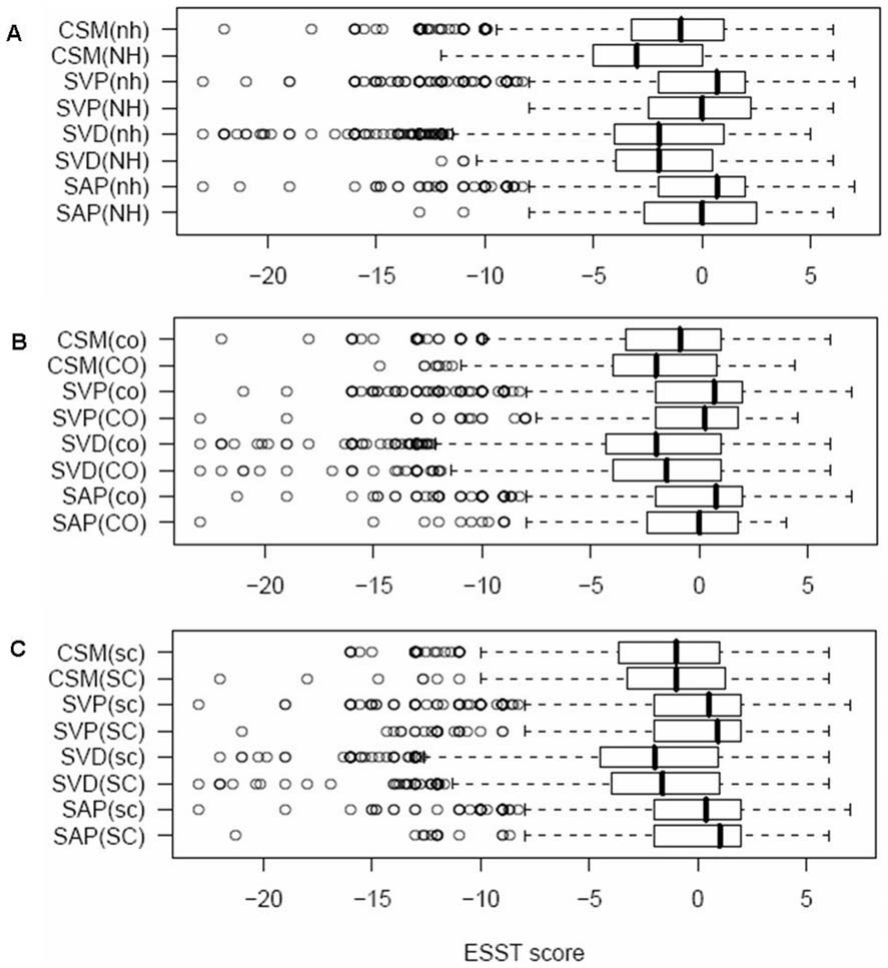
Box plots of substitution scores by hydrogen-bond types. A–C show box plots of substitution scores for the three hydrogen-bond types from a side chain: hydrogen bonds to amides (NH/nh), to carbonyls (CO/co), and to other side chains (SC/sc). The existence and absence of hydrogen bonds are shown in upper and lower case, respectively. The representation scheme of a box plot is the same as shown in Figure 1.

Figure 3 shows box plots for the distributions of substitution scores by existence or absence of hydrogen bonds from a side chain to a main-chain amide (Figure 3A), main-chain carbonyl (Figure 3B), and other side chains (Figure 3C). Overall, most of the median substitution scores for the residues making hydrogen bonds (NH/CO/SC) are smaller or equal to those from non-hydrogen bonding residues (nh/co/sc), which implies it would be more deleterious if variants were to occur at amino acids making hydrogen bonds. Indeed, the median values of SVD and CSM are negative for all three types of hydrogen bonds, although the difference is significant (*P*<1.0^−3^) only for amide (NH/nh) and carbonyl (CO/co) types of CSM dataset.

#### By elements of secondary structure

**Figure 4:**
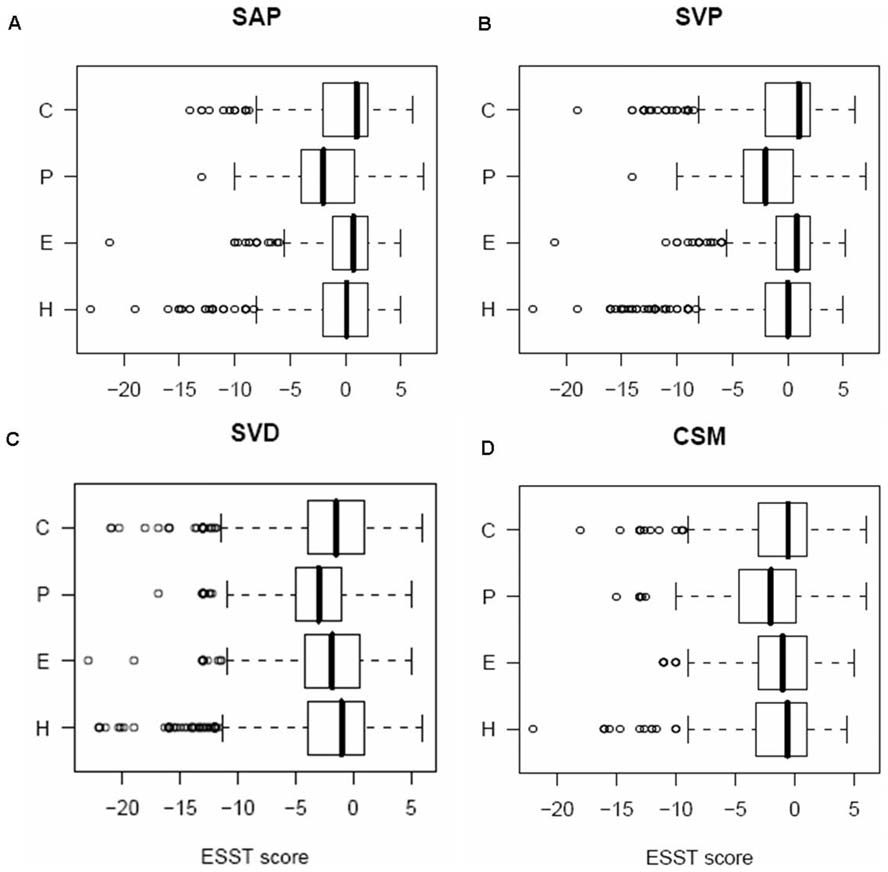
Box plots for the substitution scores by the class of secondary structure. A–D show box plots of substitution scores from four variant dataset (see Table 1) which are further divided by the element of secondary structures; α-helix (H), β-strand (E), coil (C) and residue with positive ϕ main-chain torsion angle (P). The representation scheme of a box plot is same as shown in Figure 1.

In Figure 4, we plot substitution scores by class of secondary structure at the position where the variants occur. For SVD (Figure 4C) and CSM (Figure 4D), the median values are less than zero, regardless of secondary structures. Interestingly, for all variant types, those that occur at positive ϕ main-chain torsion angles (P) are always negative and they are significantly different (*P*<1.0^−5^) from the distributions of substitution scores for helix (H), beta (E) and coil (C). A positive ϕ torsion angle can be accommodated by a Gly, which has no side chain, but for most other L-amino acids it leads to disallowed interactions between side-chain and main-chain atoms. However, for L-amino acids such as Asp or Asn, interactions between the side-chain carbonyl group with the carbonyl of the main-chain peptide bond can give rise to relative stabilisation of a conformation with a positive ϕ angle [33]. Hence, sequence variants occurring at the residues within a positive ϕ torsion angle could be very deleterious and affect the native structures. For a positive ϕ torsion angle, we found that 55∼57% of polymorphic variants (SVP and SAP) involve substitutions of amino acids from Gly, Asp and Asn, compared to 65∼68% of SVD and CSM. This suggests that disease-causing mutations affect the native structure more frequently than neutral polymorphic variants (Table S2).

### Amino Acid Property Substitution Matrix

**Figure 5:**
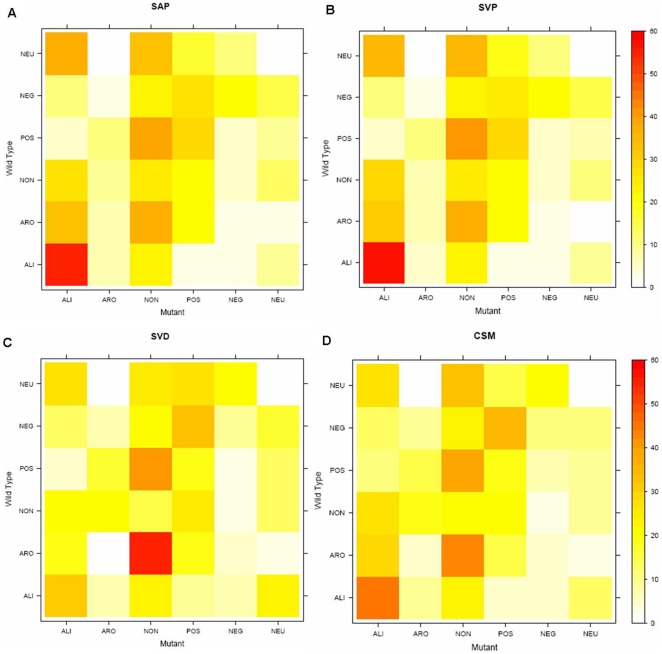
Amino acid property substitution matrices represented by heat maps. 20 amino acids are classified into six types based on their physicochemical properties (see Materials and Methods) and the substitution probabilities among the six types are represented as heat maps. A–D are from the four variant datasets in Table 1. (ALI: aliphatic, ARO: aromatic, NON: polar non-charged, POS: positively charged, NEG: negatively charged, and NEU: neutral).

Substitution scores could be a proxy for the effect of variants, but do not provide any details of amino acid substitution types. To investigate this, we classified 20 amino acids into six types on the basis of physicochemical properties of amino acids (see Material and Methods) and made 6 * 6 amino acid property substitution matrices by counting the number of substitutions of amino acid by their types. Figure 5 shows amino acid property substitution matrices for the four types of variants in which the probability of substitutions is represented as heat maps. Aliphatic amino acids (Ala, Ile, Leu, Val and Met) from SVD (Figure 5C) and CSM (Figure 5D) are relatively less conserved than those observed from SAP (Figure 5A) and SVP (Figure 5B). In addition, amino acid substitutions from negatives (Asp and Glu) to positives (Arg, His and Lys) and aromatic (Phe, Trp, and Tyr) to polar non-charged (Cys, Asn, Gln, Ser and Thr) types are more frequently observed in SVD and CSM than those observed in SAP and SVP. In terms of substitution patterns, SVP and SAP are most similar, followed by SVD and CSM, whereas SVP and SVD are most different (Table S3).

### Degree of Sequence Conservation at the Variant Locations

**Figure 6:**
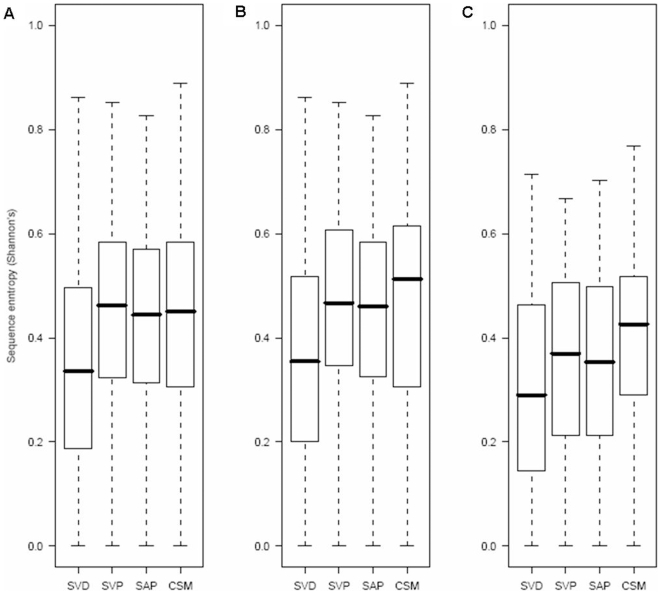
Box plots for the degree of sequence conservation measured by Shannon's entropy. Sequence entropies (see Material and Methods) from the four variant datasets (Table 1) are shown as box plots in A. Sequence entropies are calculated separately according to solvent accessibility of the variants defined by where they occur in three-dimensional structures: solvent accessible (B) and inaccessible (C). The representation scheme of the box plots is the same as shown in Figure 1.

We investigated the relationship between the variant types and the degree of sequence conservation at the locations where variants occur. Figure 6 shows box plots for the degree of sequence conservation measured by the Shannon's entropy (see Materials and Methods) from the four types of variants. In Figure 6A, it is very clear that Mendelian disease-related variants (SVD) occur at positions where amino acids are relatively conserved compared with those from polymorphic datasets (SVP and SAP) and cancer somatic mutations (CSM) with significant differences in the distribution (*P*<1.0^−11^). From Table 2, we observed that the frequency of solvent inaccessible residues is much higher for SVD than those from SVP, CSM and SAP. Hence, the lower sequence entropy of SVD might arise from relatively larger fraction of solvent inaccessible residues compared with the other variants, as solvent inaccessible residues are more conserved than solvent accessible residues. To address this issue, variants are classified into either solvent accessible (Figure 6B) or inaccessible environments (Figure 6C) and their sequence entropies were measured differently. We found that, regardless of their solvent accessibility, SVD occur at relatively conserved regions compared with variants from SVP, SAP and CSM (*P*<1.0^−7^ and *P*<0.0496 from Figure 6B and 6C, respectively). Interestingly, as shown in Figure 6B and 6C, the median entropy value of CSM is higher than that of SVP and SAP, even though the distribution is not significantly different from that of polymorphic variants (*P-values* are <0.8071, <0.7032 and <0.1240 from Figure 6A, 6B and 6C, respectively). This observation contrasts with a current report that cancer-related mutations are frequently found at evolutionarily conserved amino acid residues whereas polymorphic variants occur in relatively less conserved regions [34]. We suspect that the conflict in this observation arises from differences in the nature of the ‘cancer datasets’; we use the COSMIC database whereas the report is based on curated lists of cancer mutations from selected literatures.

### Functional Restraints

It is well understood that amino acids responsible for specific functions of proteins tend to be conserved throughout evolution and are likely to be under strong restraints. Hence, mutations that do not improve or change function in a way that confers any selective advantage to the organism would likely be deleterious. To test this, we investigated variants occur at amino acid residues responsible for protein function. We used eight functional feature types defined by UniProt annotations − ACT_SITE, BINDING, CA_BIND, DISULFID, DNA_BIND, LIPID, METAL, and NP_BIND (see Material and Methods for details) − and protein-protein interaction information from PICCOLO database (GR. Bickerton, unpublished). Table 3 shows frequencies of functional residues having a sequence variant at the position. Polymorphic variants (SVP and SAP) occur in less than 1% of functional residues, whereas Mendelian disease-related variants (SVD) occur from 1.47% for calcium-binding residues (CA_BIND) up to 10.47% for residues interacting with a metal ion (METAL). Cancer somatic mutations (CSM) occur less frequently than SVD for all functional categories, but more frequently than polymorphic variants except for two categories: BINDING (binding sites for chemical groups) and CA_BIND (calcium-binding regions).

**Table 3:** Proportion (%) of functional residues having at least one sequence variant.

Functional categories^1^	Types of variants			
	SVD^2^	SVP^3^	CSM^4^	SAP^5^
DNA_BIND	4.65	0.31	2.00	0.29
DISULFID	6.52	0.10	0.20	0.13
NP_BIND	3.91	0.25	1.39	0.32
METAL	10.47	0.21	1.16	0.18
BINDING	10.43	0.52	0.29	0.63
ACT_SITE	7.24	0.30	0.72	0.36
CA_BIND	1.47	0.54	0.22	0.51
PPI	3.53	0.83	2.15	0.51

1 : see Materials and Methods for definitions.

2 : see Dataset S2.

3 : see Dataset S4.

4 : see Dataset S8.

5 : see Dataset S6.

**Figure 7:**
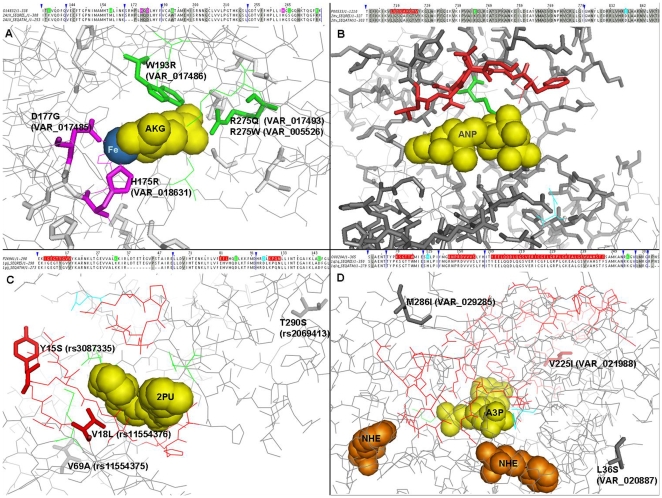
Examples of amino acid variations from the four datasets. UniProt feature annotations are transferred onto three-dimensional structures of proteins by aligning UniProt sequences with their corresponding PDB sequences using double-map method [51] (see Materials and Methods): O14832 with 2a1x in A, P00533 with 2itv in B, P24941 with 1gij in C, and O00204 with 1q1q in D. The regions not shown in the alignments are indicated with blue arrows. Amino acid variants are shown within boxes of grey background in the alignments and as bold-frame in the structure images. Metals and ligands are illustrated as spheres. Metal-binding (METAL), ligand-binding (BINDING), nucleotide phosphate-binding (NP_BIND), and active sites (ACT_SITE) residues are coloured in magenta, orange, red and cyan, respectively, both in the alignments and structure images. All structure images and alignments are drawn using PyMOL [56] and Jalview [57], respectively. (AKG: 2-Oxyglutaric acid, Fe: Iron ion, ANP: Phosphoaminophosphonic acid-adenylate ester, 2PU: 1-(5-oxo-2,3,5,9b-tetrahydro-1h-pyrrolo[2,1- a]isoindol-9-yl)-3-(5-pyrrolidin-2-yl-1h - pyrazol-3-yl)-urea, A3P: Adenosine-3′-5′-diphosphate, NHE: 2-[n-cyclohexylamino] ethane sulfonic acid).

In order to illustrate these features, we examined a number of specific cases. As an example, Figure 7 exemplifies amino acid variants occurring at functional residues mentioned above from the following four UniProt entries: O14832, P00533, P24941, and O00204 for A–D, respectively. In Figure 7A, there are 17 sequence variants annotated by UniProt, one of which (VAR_050528) is annotated as polymorphic (SVP) and the rest are disease-related variants (SVD) responsible for Refsum disease (RD) [35]–[37]. Amongst 16 disease-related variants, two occur at metal-binding (METAL) and two at ligand-binding (BINDING) residues, which are directly responsible for the disease by inducing the loss of activity for the protein [35], [37], [38]. Figure 7B illustrates the locations of cancer somatic mutations occurring at the kinase domain of EGFR (Epidermal Growth Factor Receptor). There are 10 ATP-binding sites and one active site residue of which 8 ATP-binding sites are reported amongst somatic mutations responsible for lung cancer. Figures 7C and 7D show variants in a protein kinase 2 (CDK2) and an alcohol sulfotranferase (SULT2B1), respectively. Two polymorphic variants (Y15S and V18L) occur amongst 19 ATP-binding residues in Figure 7C and only one polymorphic variant (V225I) out of 53 adenosine diphosphate binding residues in Figure 7D. The full list of all individual variants mentioned above is available as Datasets S2, S4 and S6.

### Concluding Remarks

In this report, we show that the occurrence of amino acid variants is affected by the structural and functional restraints. Based on the frequency of their occurrence in particular structural environments, disease-related variants occur more often at solvent inaccessible regions, and at amino acid residues making hydrogen bonds compared with polymorphic variants. Overall, substitution scores of Mendelian disease and cancer somatic mutations are lower than those of polymorphic variants, suggesting deleterious and harmful effects when they occur. However, we observe that there are polymorphic variants that have very low substitution scores, especially variants changing the physicochemical properties of amino acids. Indeed, the presence of polymorphic variants (SVP and SAP) in our dataset does not necessarily mean they are neutral with respect to the phenotypes. There are likely to be variants related to a certain disease type, which have not been identified yet. However, we did not attempt to predict sequence variants causing deleterious effects on protein structures and depriving functions, which eventually lead to a specific disease, as they have been addressed extensively by others [39]–[43]. Rather, we focused on the distributions and occurrences of amino acid variants in terms of structural and functional features of proteins.

Cancers arise from mutations in a subset of genes that confer growth advantage to the tumour. However, our analysis, based on substitution scores and amino acid property matrices, showed that the severity of cancer somatic mutations lies between that of Mendelian disease-related variants and polymorphic variants; less deleterious than Mendelian disease causing variants but more severe than polymorphic variants. Recently, Talavera *et al.* [34] investigated the pattern of cancer-related mutations and compared them with those from polymorphic variants. They showed that the distribution of cancerous amino acid substitutions is very similar to that of polymorphism, suggesting they are under similar selection pressures by neutral evolution, although polymorphic variants tend to occur at less conserved positions than cancer-related mutations. It is known that not all somatic mutations confer growth advantage to the cells. There are ‘driver’ somatic mutations which are the main contributors to the development of the cancers, whereas most somatic point mutations are likely to be ‘passengers’ that do not contribute to oncogenesis [44]. However, it is not a trivial problem discriminating between the two and our dataset almost certainly contains both types, obscuring the effect of ‘driver’ mutations.

At the time of this study, reported SNPs comprise 0.46% (0.13% for verified SNPs) of the total number of human DNA base pairs of which 53% of SNPs occur at intergenic regions and 36% occur at intronic region (Table S4). Only 1.26% of human SNPs occur in protein coding regions in which more than half are non-synonymous SNPs (0.64%) – those that have been considered in the study – and the rest are synonymous SNPs (0.46%), frame shift (0.09%) and stop gained mutations (0.02%). Throughout our analysis we did not take the expression level into account; rather we assumed that proteins are equally expressed no matter whether they contain sequence variants or not. However, it is clear that proteins having deleterious mutations are selectively controlled by the protein degradation system to protect against misfolded or damaged proteins [45] and sometimes those mutations are compensated in other species [46].

## Materials and Methods

### Variants Data Source

SVD and SVP are defined by annotations of UniProt human sequence variations (http://www.uniprot.org/docs/humsavar.txt, release: 57.5) where types of amino acids variants are classified either disease, polymorphism or unclassified [23]. For SVD, variants are further filtered out by removing non-Mendelian diseases which have not been assigned any MIM number from the OMIM (http://www.ncbi.nlm.nih.gov/omim/) database and any disease names related with cancers from the following key tokens: cancer, tumor, neoplasia, leukaemia, lymphoma, melanoma, carcinoma, blastoma, and cytoma. CSM is taken from the COSMIC (Catalogue of Somatic Mutation in Cancer, http://www.sanger.ac.uk/genetics/CGP/cosmic/, version: 42) database [25] from which mutations result in amino acid changes were taken and SAP is from the Ensembl human variation database (http://www.ensembl.org, database version: 54_36p) [24] which compiles SNPs (Single Nucleotide Polymorphisms) mainly from dbSNP database (http://www.ncbi.nlm.nih.gov/projects/SNP/) [47]. From Ensembl human variations, we have used only verified SNPs; genotyped and validated by the international HapMap project [48]. Amino acid variants of CSM and SAP were transferred onto the positions of their corresponding UniProt sequence using the sequence alignment program, BL2SEQ, of NCBI blast package [49] if necessary.

### Representative SCOP Domains

SCOP 1.71 was used to define representative domains by applying the following conditions:

1. NMR structures and proteins having resolution worse than 2.5 Å were excluded.
2. Protein domains were clustered for each SCOP family by running CD-HIT [50] with sequence identity of 80% or more.
3. Within a SCOP family, the average sequence length is maintained by removing any domains having sequence below of (1-0.3)*mean-length and above of (1+0.3)*mean-length.
4. Within a cluster, a protein structure having the best resolution was selected as a representative.

Non-canonical SCOP classes (H, I, J, and K,) and membrane and cell surface proteins (F) were not included in the process described above.

### Mapping the Location of Variants onto Three-Dimensional Structure

To locate the position of a sequence variant in the three-dimensional structure, variants mapped onto UniProt sequences were further transferred onto three-dimensional structures using double-map [51] which aligns a sequence of UniProt to its corresponding PDB structure at residue level. In short, double-map makes two alignments from the three sequences. The first alignment is between a sequence in atomic coordinate record (SEQATM) and SEQRES record of a PDB file. The second is between SEQRES and its corresponding UniProt sequence (SP). Using SEQRES as a reference SP can be aligned with SEQATM and the locations of UniProt residues can be mapped onto three-dimensional structures.

### Identifying Local Structural Environment of Amino Acids

We used JOY [52] to identify the local structural environments of amino acids. JOY consists of three supporting programs − SSTRUC, PSA, and HBOND − to annotate 1) the elements of secondary structure, 2) solvent accessibility, 3) hydrogen bonds from side chains, respectively. SSTRUC calculates torsion angles within a main chain to assign secondary structure. For the threshold of solvent accessibility, we used a cut-off of 7.0% relative total side-chain accessibility. HBOND identifies all possible hydrogen bonds based on a distance criterion; 3.5 Å between donor and acceptor except for interactions involving sulphur atoms where 4.0 Å is used.

### Amino Acid Substitution Scores

For variants at the UniProt protein sequence level, we looked up BLOSUM62 [32] to get the substitution score for a corresponding variant. However, substitution scores for the variants mapped onto three-dimensional structures were from an Environment Specific Substitution Table (ESST) [28], [29] which corresponds to the local structural environment for a variant. We used ALL-B type of ESST, which has proved to be the best in the previous benchmarking tests [51]. The detailed procedure of making ESSTs is explained in our recent paper and the ESST web site (http://www-cryst.bioc.cam.ac.uk/ESST). ESST can be generated in an automatic fashion by a recently developed computer software Ulla [53].

### Statistical Analysis

Wilcoxon rank sum test were used to calculate significant difference in the distribution of substitution scores between two groups. We used *wilcox.test* of *stats* package of R [54] with a two-sided test option.

### Classification of Amino Acid Types

20 amino acids are classified into 6 classes by their physicochemical properties as follows:

1. Aliphatic (ALI): Ala, Ile, Leu, Val and Met
2. Aromatic (ARO): Phe, Trp, and Tyr
3. Polar non-charged (NON): Cys, Asn, Gln, Ser and Thr
4. Positively charged (POS): Arg, His and Lys
5. Negatively charged (NEG): Asp and Glu
6. Neutral (NEU): Gly and Pro

### Sequence Entropy

To measure the degree of sequence conservation, we calculated sequence entropy for each alignment position within a protein family having at least three sequences. We skipped measuring entropy if gaps occur in more than 50% of sequences at the alignment position. We used Shannon's entropy equation [55] which can be formulated as below:
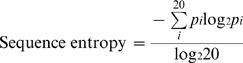
where *pi* is the frequency of amino acid *i* (of 20) at the alignment position.

### Definitions of Functional Residues

Variants taken from the four types of dataset were examined to see whether they occur at protein residues responsible for specific functions. We defined functional residues if they are annotated by UniProt functional features (from ‘FT’ lines) or known to maintain protein interactions detected by PICCOLO (GR. Bickerton, unpublished) which is an in-house database of protein-protein interactions between every pair of chains from protein structures in PDB. We used eight types of UniProt functional features:

1. ACT_SITE: amino acid(s) involved in the activity of an enzyme
2. BINDING: binding site for any chemical group (e.g. co-enzyme, prosthetic group, etc.)
3. CA_BIND: extent of a calcium-binding region
4. DISULFID: disulfide bonds
5. DNA_BIND: extent of a DNA-binding region
6. LIPID: covalent binding of a lipid moiety
7. METAL: binding site for a metal ion
8. NP_BIND: extent of a nucleotide phosphate-binding region

## Supporting Information

Figure S1 A Venn diagram showing the number of overlaps amongst variant datasets.
(0.03 MB DOC)

Table S1 Ratios of variants having negative and non-negative substitution scores.
(0.06 MB DOC)

Table S2 Percentage (%) of amino acid variants occurring at positive ϕ main-chain torsion angle.
(0.05 MB DOC)

Table S3 Distance matrix of amino acid mutations from the four types of variants.
(0.03 MB DOC)

Table S4 Total number of SNPs by different types of their consequences.
(0.04 MB DOC)

Dataset S1 Annotations of structural environments for SVD dataset.
(2.16 MB ZIP)

Dataset S2 Function annotations for SVD dataset.
(0.29 MB TXT)

Dataset S3 Annotations of structural environments for SVP dataset.
(2.53 MB TXT)

Dataset S4 Function annotations for SVP dataset.
(0.04 MB TXT)

Dataset S5 Annotations of structural environments for SAP dataset.
(1.05 MB TXT)

Dataset S6 Function annotations for SAP dataset.
(0.03 MB TXT)

Dataset S7 Annotations of structural environments for CSM dataset.
(10.63 MB TXT)

Dataset S8 Function annotations for CSM dataset.
(0.04 MB TXT)
