# Supplementary material for: Structural and Functional Restraints on the Occurrence of Single Amino Acid Variations in Human Proteins
Source: PLoS One. 2010 Feb 12;5(2):e9186. doi: 10.1371/journal.pone.0009186 (PMC2820541; doi:10.1371/journal.pone.0009186)
Supplement: Figure S1 — A Venn diagram showing the number of overlaps amongst variant datasets. (0.03 MB DOC) [file pone.0009186.s001.doc]

# Supplementary Figures

Figure S1. A Venn diagram showing the number of overlaps amongst variant datasets.

**SVP**

16442

12569

15862

4208

2

8

5

230

16855

23

102

**SVD**

**CSM**

**SAP**

Four variants datasets (SVD, SVP, SAP and CSM) are from Table 1. (SVD: Mendelian disease-related variants, CSM: Cancer somatic mutations, SVP and SAP: Polymorphic variants, see ‘Compilation of amino acid variant dataset’ of Results and Discussion section)
