# Supplementary material for: Structural and Functional Restraints on the Occurrence of Single Amino Acid Variations in Human Proteins
Source: PLoS One. 2010 Feb 12;5(2):e9186. doi: 10.1371/journal.pone.0009186 (PMC2820541; doi:10.1371/journal.pone.0009186)
Supplement: Table S1 — Ratios of variants having negative and non-negative substitution scores. (0.06 MB DOC) [file pone.0009186.s002.doc]

# Supplementary Tables

Table S1. Ratios of variants having negative and non-negative substitution scores.

| Structural environment | | | | | Types of variants | | | | | | | | | | |
| --- | --- | --- | --- | --- | --- | --- | --- | --- | --- | --- | --- | --- | --- | --- | --- |
| categories | | | types | | SVD | | SVP | | | | CSM | | SAP | | |
|  |  |  |  |  | <0 | >=0 | <0 | | | >=0 | <0 | >=0 | <0 | | >=0 |
| Solvent accessibility | | | A^1^ | | 0.58 | 0.42 | 0.38 | | | 0.62 | 0.51 | 0.49 | 0.37 | | 0.63 |
|  |  |  | a^2^ | | 0.70 | 0.30 | 0.36 | | | 0.64 | 0.67 | 0.33 | 0.41 | | 0.59 |
| Hydrogen bonds from sidechains | to main-chain amide | | F^3^ | | 0.62 | 0.38 | 0.37 | | | 0.63 | 0.54 | 0.46 | 0.38 | | 0.62 |
|  |  |  | T^4^ | | 0.7 | 0.3 | 0.45 | | | 0.55 | 0.67 | 0.33 | 0.43 | | 0.57 |
|  | to main-chain carbonyl | | F | | 0.63 | 0.37 | 0.37 | | | 0.63 | 0.53 | 0.47 | 0.37 | | 0.63 |
|  |  |  | T | | 0.63 | 0.37 | 0.38 | | | 0.62 | 0.64 | 0.36 | 0.43 | | 0.57 |
|  | to other side chains | | F | | 0.63 | 0.37 | 0.37 | | | 0.63 | 0.54 | 0.46 | 0.38 | | 0.62 |
|  |  |  | T | | 0.62 | 0.38 | 0.39 | | | 0.61 | 0.56 | 0.44 | 0.37 | | 0.63 |
| secondary structure | | | H^5^ | | 0.59 | 0.41 | 0.4 | | | 0.6 | 0.52 | 0.48 | 0.4 | | 0.6 |
|  |  |  | E^6^ | | 0.65 | 0.35 | 0.3 | | | 0.7 | 0.58 | 0.42 | 0.35 | | 0.65 |
|  |  |  | P^7^ | | 0.79 | 0.21 | 0.62 | | | 0.38 | 0.68 | 0.32 | 0.62 | | 0.38 |
|  |  |  | C^8^ | | 0.61 | 0.39 | 0.35 | | | 0.64 | 0.52 | 0.48 | 0.35 | | 0.65 |
| All | | | | | 0.63 | 0.37 | 0.37 | | | 0.63 | 0.55 | 0.45 | 0.38 | | 0.62 |
| ^1^: accessible | | ^2^: inaccessible | | ^3^: False (no hydrogen bonds) | | | | ^4^: True (hydrogen bonded) | | | | | |  | |
| ^5^: α-helix | | ^6^: β-strand | | ^7^: positive φ main-chain torsion angle | | | | | ^8^: coil | | | | | | |
