# Supplementary material for: Structural and Functional Restraints on the Occurrence of Single Amino Acid Variations in Human Proteins
Source: PLoS One. 2010 Feb 12;5(2):e9186. doi: 10.1371/journal.pone.0009186 (PMC2820541; doi:10.1371/journal.pone.0009186)
Supplement: Table S2 — Percentage (%) of amino acid variants occurring at positive ϕ main-chain torsion angle. (0.05 MB DOC) [file pone.0009186.s003.doc]

# Supplementary Tables

Table S2. Percentage (%) of amino acid variants occurring at positive φ main-chain torsion angle.

| Wild type Amino acids | SVD | SVP | SAP | CSM |
| --- | --- | --- | --- | --- |
| G | 58.59 | 42.64 | 44.06 | 55.65 |
| R | 6.11 | 11.68 | 13.29 | 6.09 |
| N | 4.20 | 7.11 | 7.69 | 2.17 |
| A | 4.01 | 3.55 | 1.40 | 0.43 |
| D | 3.24 | 6.60 | 7.69 | 6.96 |
| S | 2.86 | 5.58 | 4.90 | 5.22 |
| C | 3.63 | 1.02 | 3.50 | 0.00 |
| E | 2.29 | 3.55 | 3.50 | 4.35 |
| F | 1.91 | 1.02 | 1.40 | 0.00 |
| M | 2.67 | 0.00 | 0.00 | 1.74 |
| L | 2.48 | 2.54 | 2.10 | 1.30 |
| Y | 1.72 | 1.02 | 1.40 | 1.30 |
| K | 0.76 | 3.05 | 2.10 | 3.91 |
| Q | 1.72 | 2.54 | 2.10 | 1.30 |
| T | 1.53 | 1.02 | 0.70 | 0.43 |
| H | 0.95 | 3.05 | 2.10 | 3.48 |
| V | 0.76 | 1.52 | 0.70 | 0.87 |
| I | 0.38 | 0.51 | 0.00 | 3.04 |
| W | 0.19 | 1.52 | 0.70 | 0.00 |
| P | 0.00 | 0.51 | 0.70 | 1.74 |
