# Supplementary material for: Structural and Functional Restraints on the Occurrence of Single Amino Acid Variations in Human Proteins
Source: PLoS One. 2010 Feb 12;5(2):e9186. doi: 10.1371/journal.pone.0009186 (PMC2820541; doi:10.1371/journal.pone.0009186)
Supplement: Table S3 — Distance matrix of amino acid mutations from the four types of variants. (0.03 MB DOC) [file pone.0009186.s004.doc]

# Supplementary Tables

Table S3. Distance matrix of amino acid mutations from the four types of variants.

|  | CSM | SAP | SVD |
| --- | --- | --- | --- |
| SAP | 31.11 |  |  |
| SVD | 26.72 | 43.86 |  |
| SVP | 32.21 | 6.54 | 45.26 |

The Euclidean distance ($\text{DIST(X}\cdot\text{ Y)}$), between two amino acid property substitution matrices, X and Y, defined as;

$\mathrm{DIST}(\text{X}\cdot\text{Y})=\left(\sum_{j=1}^{6} \sum_{k=1}^{6} \left(X_{j\to k}-Y_{j\to k}\right)^{2}\right)^{1/2}$where $X_{j\to k}$ and $Y_{j\to k}$ are the probabilities of amino acid category *j* to be substituted by category *k* from the variant dataset X and Y, respectively.
