# Supplementary material for: Structural and Functional Restraints on the Occurrence of Single Amino Acid Variations in Human Proteins
Source: PLoS One. 2010 Feb 12;5(2):e9186. doi: 10.1371/journal.pone.0009186 (PMC2820541; doi:10.1371/journal.pone.0009186)
Supplement: Table S4 — Total number of SNPs by different types of their consequences. (0.04 MB DOC) [file pone.0009186.s005.doc]

# Supplementary Tables

Table S4. Total number of SNPs by different types of their consequences.

| type | num | Ratio (%) |
| --- | --- | --- |
| INTERGENIC | 7,982,768 | 53.07 |
| INTRONIC | 5,481,863 | 36.45 |
| UPSTREAM | 663,985 | 4.41 |
| DOWNSTREAM | 556,742 | 3.70 |
| 3PRIME_UTR | 137,639 | 0.92 |
| NON_SYNONYMOUS_CODING | 96,031 | 0.64 |
| WITHIN_NON_CODING_GENE | 86,955 | 0.58 |
| SYNONYMOUS_CODING | 69,035 | 0.46 |
| 5PRIME_UTR | 28,343 | 0.19 |
| FRAMESHIFT_CODING | 14,002 | 0.09 |
| REGULATORY_REGION,INTRONIC | 13,365 | 0.09 |
| SPLICE_SITE,INTRONIC | 10,457 | 0.07 |
| REGULATORY_REGION,UPSTREAM | 4,951 | 0.03 |
| REGULATORY_REGION,INTERGENIC | 4,949 | 0.03 |
| NON_SYNONYMOUS_CODING,SPLICE_SITE | 2,845 | 0.02 |
| STOP_GAINED | 2,533 | 0.02 |
| data from Ensemble human variations |  |  |
